# A flexible polypyrrole/GelMA self-supported electrode for supercapacitors by confined interfacial electrodeposition

**DOI:** 10.1039/d5ra08151c

**Published:** 2026-01-19

**Authors:** Gang Wang, Xuezheng Chen, Jiafan Guo, Xiaomin Su, Weijie Li, Junyi Huang, Wenxi Wu, Ting Tian, Feng Yu

**Affiliations:** a School of Materials,Guangdong Industry Polytechnic University Guangzhou 510300 China; b College of Materials and Energy, Foshan University Foshan 528225 China yuf@fosu.edu.cn; c College of Chemical Engineering, Fuzhou University Fuzhou 350116 China; d Guangdong Canosafe Pharmatech Co., Ltd Guangzhou 510300 China

## Abstract

The traditional polypyrrole (PPy) electrode for supercapacitors usually consists of PPy nanoparticles, carbon black and a binder. The PPy electrode inevitably suffers from structural rupture and active PPy particles' detachment during repeated doping/de-doping processes and results in low practical capacitance. Exploring the self-supported electrode without adding any binder or conductive agent is a smart and promising method to avoid the above problems. Herein, a flexible and tailorable polypyrrole/GelMA (PPy/GEL) composite film electrode was prepared *via* a novel confined interfacial electropolymerization at the interface of GelMA gel film and a conductive glass substrate (FTO). This method leverages spatial confinement to guide PPy growth into a continuous film, as confirmed by cross-sectional SEM analysis. This method can well confine the growth direction of the PPy chain so that PPy grows laterally to easily form a flexible and smooth two-dimensional film. The PPy/GEL-based supercapacitor not only exhibits a high areal capacitance of 260 mF cm^−2^ at a current density of 1 mA cm^−2^, which is almost 3 times higher than the pure PPy-based supercapacitor (76 mF cm^−2^), but also shows high cycling stability with 98% capacitance retention even after 30 000 cycles. Furthermore, the device demonstrates remarkable mechanical durability, retaining its electrochemical performance without significant degradation after being subjected to 100 repeated 180-degree folding cycles, underscoring its potential for wearable applications. This work provides a good strategy for the preparation of high performance PPy self-supported film electrodes.

## Abstract 2

The traditional polypyrrole (PPy) electrode for supercapacitors usually consists of PPy nanoparticles, carbon black and a binder.

## Graphical abstract

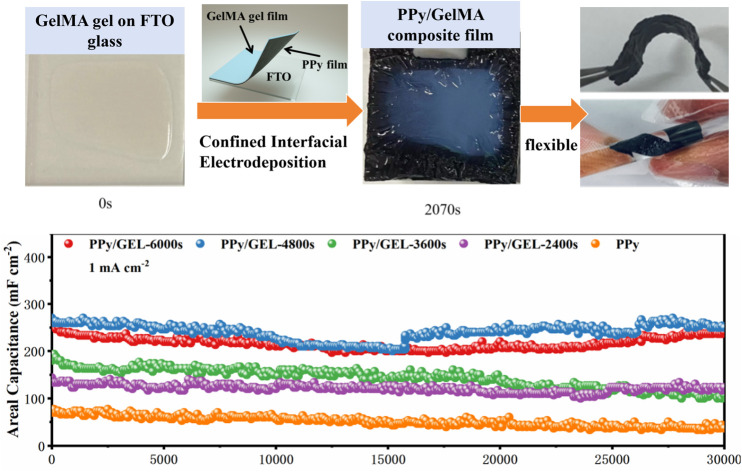

## 1. Introduction

The advancement of science and technology, particularly in flexible electronics, has driven demand for high-performance energy storage devices.^1^ Recent trends in smart wearables and electronic skin require sustainable supercapacitor solutions.^2^ Intelligent hardware, smart phones, and smart wearable devices are also the trend in recent years, especially the rapid progress of electronic technology, but also to make these electronic devices thin, diversified, and in a flexible direction, and then research and develop a foldable, flexible new generation of flexible electronic products. In the future, the popularity of smart fabrics, smart glasses, smart sports equipment, electronic skin, and flexible screens will require the support of flexible devices.^3^

Conductive polymers (CPs) are often used as active electrode materials for flexible devices.^4^ CPs, such as polypyrrole (PPy) and polyaniline (PANI), have the advantages of high specific capacitance, low cost and easy fabrication, but their relatively low mechanical stability and cycle life restrict their practical application. The main reasons for the low cycle stability of CPs are easily structural disintegration and poor conductivity.^5^

CPs is typical pseudocapacitive materials which are prone to structural disintegration. Taking polyaniline (PANI) as an example, its charge storage activity depends on the doping and de-doping of counterions during charge and discharge, respectively. PANI starts from undoped brilliant copperas, then transitions to partially doped copperas salts, and finally to fully doped peheniline salts. The insertion of the counterion pulls in the solvent molecules and also causes the polyaniline to swell.^6^ Long-term continuous and repeated charge–discharge cycles will amplify the destructive force of osmotic swelling and significantly reduce cycle stability.^5^ At the same time, the composition changes caused by the loss of counter ions, auto-oxidation, peroxidation, over-reduction and dissolution of active components also lead to poor conductivity of conductive polymers.^7^

In order to solve these two problems of CPs, three strategies are proposed: spatial confinement, surface coating and ordering molecular structure.^5^ Simply put, the spatial confinement is using mechanically flexible materials to composite with conductive polymer. Even if the structure of the conductive polymer is unstable during charging and discharging, the mechanical soft material can stabilize the structure of the conductive polymer. For example, Zhao *et al.* used vapor deposition polymerization techniques to prepare PANI coated hierarchical porous carbon (HPC) composites. The cyclic stability of the electrode reached 96.1% after 10 000 cycles of charge–discharge at 10 A g^−1^, while the pure PANI only retained 40% of the capacitance after 1000 cycles.^8^ Gogotsi *et al.* suggested that introducing hydrophilic functional groups to form covalent and/or hydrogen bonds with conjugated polymers could enhance the cycling stability of polypyrrole (PPy). They polymerized pyrrole *in situ* in the layer of MXene compound Ti_3_C_2_T_*x*_ (T_*x*_ represents the terminal functional group on the Ti–C layer), and the final Py/Ti_3_C_2_T_*x*_ composite film had a capacitance 1.5 to 2.5 times higher than that of the bare Ti_3_C_2_T_*x*_ electrode, and the capacitance retention rate after 25 000 charge and discharge cycles was 92%.^9^

Surface coating is the use of coating materials to protect the structural integrity of CPs. Lyu *et al.* infiltrated polyaniline (PANI) and graphene oxide (GO) or carboxyl multi-walled carbon nanotubes (CMCNT) into the aerogel by vacuum filtration to form aerogel electrodes using porous aerogel as substrate. The layer-by-layer (LbL) assembly of the electroactive material into the three-dimensional aerogel framework is realized, the utilization rate and the electrochemical performance of the electroactive material are greatly improved.^10^ Ali Khosrozadeha and coworkers developed a novel flexible electrode by growing polyaniline nanowires on graphene-coated polyvinyl alcohol (PVA) nanofibers and then re-coating them on the graphene flakes as size charge collectors. After 18 000 cycles at 35 mA, the retention of capacitance and power density of the electrode was 96.3% and 100%, respectively. The retention of capacitance and power density after 76 000 cycles at 50 mA is 77.9% and 98.5%, respectively.^11^

The ordered molecular structure has an effect on the cyclic stability of CPs. For example, Zhi *et al.* compared the PPy films synthesized by electrochemical oxidation and chemical oxidation, because the orientation electric field is beneficial to the deprotonation of pyrrole at α-position, resulting in PPy with planar configuration but the uniform oxidation environment of Fe^3+^ has no guiding effect, which makes the performance of the two PPy films different. They found that the planar configuration of α-carbon with α-carbon is more stable than the anisotropic configuration of PPy.^12^ Huang *et al.* uniformly grew 4-butylbenzenesulfonate modified PPy thin films on filter paper substrates. The flexible freestanding polypyrrole paper electrode has good cycling stability, and the capacitance retention rate is 84.8% after 200 thousand cycles. They believe that the modification of 4-butylbenzenesulfonate can improve the order of PPy by stacking α–α coupling chains layer by layer, make it structurally stable during charge and discharge.^13^

In this work, we improve the electrochemical performance of PPy through the strategy of spatial confinement. Compared to direct electrochemical deposition, electrochemical deposition by coating gelatin hydrogel on conductive glass (FTO) enables PPy to grow laterally within the interface between gelatin hydrogel film and conductive glass. Both the gelatin film and FTO restrict the growth of PPy, thus forming a continuous and dense composite film labelled as PPy/GEL. The composite PPy/GEL electrode has excellent electrochemical properties. The optimal PPy/GEL-4800s SC can achieve a high surface capacitance of 260 mF cm^−2^ at a current density of 1 mA cm^−2^. Furthermore, this PPy/GEL-4800s SC showed no significant capacity decline after 30 000 cycles.

## 2. Experimental section

### 2.1 Synthesis of GelMA

Briefly, 5 g of gelatin was dissolved in 100 mL of PBS (pH = 7.4) buffer and stirred in a thermostatic water bath at 60 °C until completely dissolve. Added 4 mL of methacrylic anhydride dropwise to the gelatin solution, reacted at 50 °C for 6 h, then put it into the dialysis bag, dialyzed with deionized water for 3 days (change water 2–3 times a day), and freezed dry for 3 days to obtain the product. The reaction product was denoted GelMA (GEL).

### 2.2 Synthesis of PPy/GEL

0.5 g GEL was dissolved in 5 mL of deionized water (DI) to obtain solution A. 5 mg Ir2959 was dissolved in 100 µL absolute ethanol to gain solution B. Subsequently, the above 50 µL of solution B was added into solution A. Furthermore, 200 µL mixture was painted on Conductive glass (FTO) by film applicator, and then the FTO was put into fully functional UV curing tank (wavelength 365 nm) for two minutes of light curing. Finally, in a solution containing pyrrole and 0.3 M NaClO_4_ (volume ratio 1 : 40), the electrochemical polymerization of Pyrrole was carried out on an FTO substrate by potentiostatic technology at a voltage of 0.6 V to obtain PPy/GEL. According to different reaction time, products were named PPy/GEL-6000s, PPy/GEL-4800s, PPy/GEL-3600s and PPy/GEL-2400s. For comparison, pure PPy was prepared following the same procedure but without adding GEL.

### 2.3 Assembly of symmetric supercapacitor

The PPy-GEL film was cut into electrode pieces with a diameter of 10 mm (geometric area = 0.785 cm^2^), and then the electrode pieces were pressed on carbon paper as a current collector, and finally assembled into supercapacitor.

### 2.4 Materials and characterizations

Py, Methacrylic anhydride and Gelatin were all analytical grades and purchased from Macklin. PBS buffer (0.01 M pH = 7.2–7.4) was purchased from Solarbio. NaClO_4_ is a high grade pure reagent purchased from Asahi Kasei. All aqueous solutions were prepared with deionized water (DI).

The morphologies and structural information of the materials were characterized by scanning electron microscopy (SEM, Hitachi SU8010), the UV/visible/NIR spectrophotometer (UV-vis, Hitachi UH5760), the Raman spectroscopy (Horiba Xplora plus), X-ray photoelectron spectroscopy (XPS) (Thermo Escalab 253Xi) and Four-probe resistivity tester (4Probes Tech Ltd RTS-8).

### 2.5 Electrochemical measurement and calculation

Cyclic voltammetry (CV) was conducted by Biologic VSP-300 workstation with a voltage range of 0–0.6 V. EIS measurements were tested on the Biologic VSP-300 workstation with a frequency range of 100 kHz to 0.01 Hz. Galvanostatic charge/discharge (GCD) measurements were carried out on the LAND CT 2001A system.

PPy/GEL and neat PPy films were cut into pole pieces with a diameter of 10 mm and then pressed directly onto carbon paper. Supercapacitor devices for two-electrode testing were assembled by encapsulating two PPy-GEL electrodes or pure PPy electrodes of the same deposition time. The electrolyte was 1 M H_2_SO_4_.

The specific capacitance (C) of supercapacitor device is computed by the galvanostatic charge–discharge (GCD) curve *via* the following equations:a
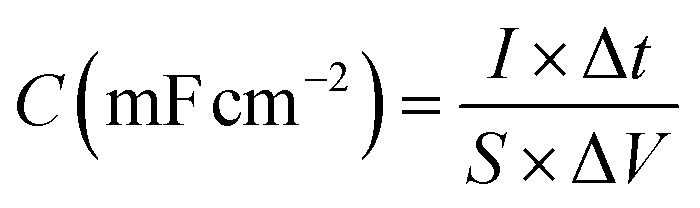
where *I* (mA), Δ*t* (s), *S* (cm^2^, geometric area = 0.785 cm^2^ for a 10 mm diameter electrode), and Δ*V*(V) are the discharging current and time, active-material area, and discharging potential window, respectively.

For a supercapacitor device, energy density (E), and power density (P) could be derived from the following equations:b
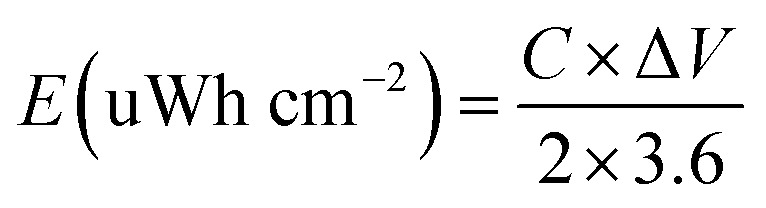
c
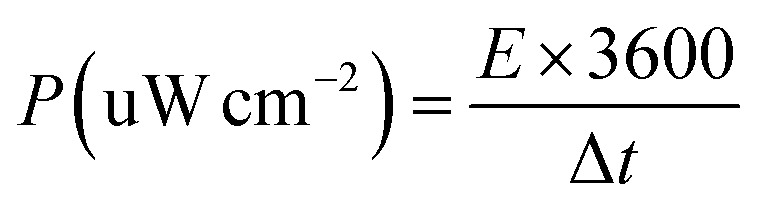


## 3. Results and discussion

### 3.1 Preparation of PPy/GEL electrodes in interfacial deposition

**Fig. 1:**
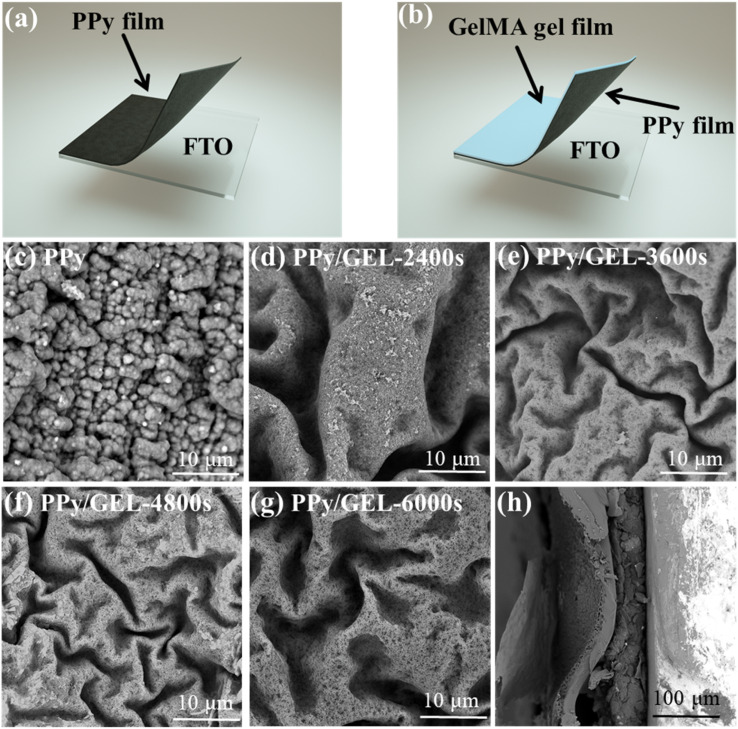
Schematic diagram of surface and interfacial electrodeposition models (a) and (b). Surface SEM morphologies of (c) PPy, (d) PPy/GEL-2400s, (e) PPy/GEL-3600s, (f) PPy/GEL-4800s and (g) PPy/GEL-6000s. (h) Cross-sectional SEM image of the PPy/GEL-4800s.

Fig. 1a shows a schematic diagram for direct electrodeposition of PPy electrodes. Fig. 1b displays the preparationfor the PPy/GEL electrodes. GelMA precursor solution is coated in FTO through film applicator, and then the Py monomer is polymerized to form polypyrrole between the GEL and FTO by a three-electrode system. The growth of PPy would be limited because of the existence of GelMA. Finally, the obtained PPy/GEL film can be easily torn off from FTO. The PPy/GEL films with various deposition time are shown in Fig. S1. It is very obvious that it takes 2070s to form a complete PPy/GEL film.

The obtained PPy/GEL film possesses certain flexibility and cutting ability (Fig. 3e), that it can be bent and cut into various letters. Furthermore, in terms of visualization, the PPy/GEL strip can be used directly as flexible conductors in circuits containing a 4.5 V power supply to power the blue LED bulb (Fig. 3e). To quantitatively evaluate the mechanical durability, the PPy/GEL-4800s electrode was subjected to 100 repeated 180-degree folding cycles. As shown in Fig. S2, the capacitive performance of the electrode remained almost unchanged after this rigorous folding test, providing strong evidence for its exceptional mechanical and electrochemical robustness under deformation. Moreover, without sacrificing their conductivity, these stripes can be bent into different shapes, such as arc-shape and S-shape.

The morphological evolution of PPy during direct deposition and interfacial deposition are investigated by SEM. In Fig. 1c, the directly deposited PPy film is composed of many obvious polypyrrole particles. On the contrary, the PPy film deposited at the interface is a continuous and compact film (Fig. 1d–g). From the perspective of surface energy, these nanoparticles have a higher surface energy than nanofilms and tend to be unstable.^12^Fig. 1h exhibits the cross-sectional morphology of the PPy/GEL-4800s sample, indicating that PPy and GEL are clearly stratified and closely bound. The PPy layer has a thickness of several hundred nanometers to approximately 1 µm, while the GelMA layer is several micrometers thick.

### 3.2 Characterizations of PPy/GEL electrodes

**Fig. 2:**
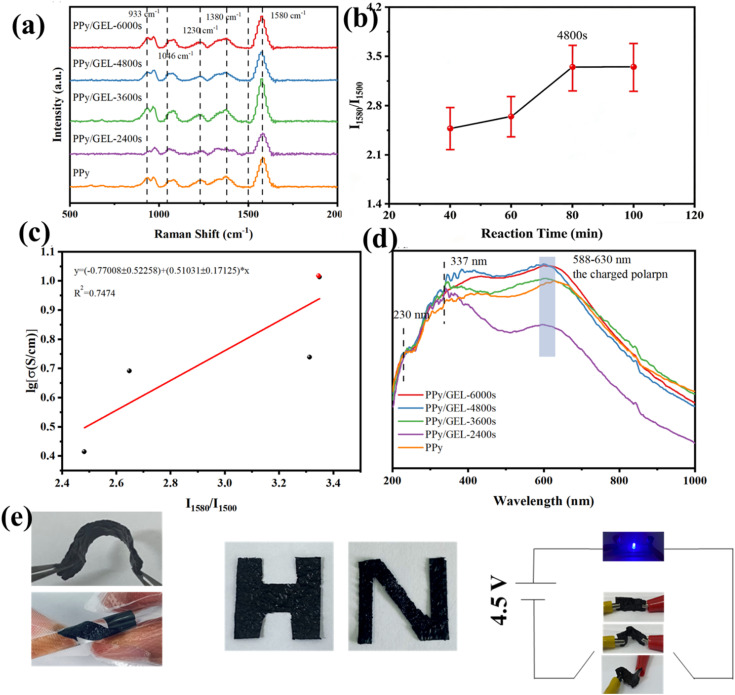
(a) Raman spectra of PPy films grown with different deposition time and pure PPy, (b) plot of the conjugation length and the reaction time, (c) linear fitting plot of the conjugation length and the electrical conductivity and (d) UV-vis absorption spectra of PPy film grown with different deposition time and pure PPy. (e) The PPy/GEL film is flexible and can be cut into various letters and PPy/GEL stripe under various deformations can be used as a conductor in a circuit for lighting blue LED lamp.

The Raman measurement was used to further study the molecular vibration of the pure PPy and PPy/GEL samples. From the survey spectra in Fig. 2a, all samples showed a similar characteristic PPy peak. The peak value is approximately 1580 cm^−1^, which is due to the main chain stretching mode of the C=C bond. A wide ring stretching mode^14^ is observed at 1380 cm^−1^. The most significant peak is noticed in 1230 cm^−1^, which is due to N–H in planar bending mode and is the finger print of PPy.^15^ In addition, two more peaks^16^ were found which represent C–H in plane deformation at 933 cm^−1^ to 1046 cm^−1^. Furthermore, the intensity ratio of Raman bands of the symmetric C=C stretching mode at 1580 cm^−1^ to the skeletal band at 1500 cm^−1^ can also be used to determine the relative conjugation length, and the intensity ratio of the two bands (*I*_1580_/*I*_1500_) increase with the increase of the conjugation length.^17^ As shown in Fig. 2b, the conjugation length of PPy/GEL becames longer with the increase of reaction time, but the conjugation length of PPy/GEL-4800s was similar to that of PPy/GEL-6000s, which might be because the loading of active substance on PPy/GEL-4800s is similar to that on PPy/GEL-6000s.

The electrical conductivities of PPy and PPy/GEL were determined using the RTS-4 four point probe resistivity measurement system. And the linear relationship can also be found in Fig. 2c and Table S1, where lg[*σ*] is a function of *I*_1580_/*I*_1500_. The linear fitting index is 0.9376 that is approach to 1. The results verify that the higher conjugate length of PPy chain results in good electric conductivity of PPy film. From the data showed in Table S1, the electric conductivity of PPy/GEL-6000s is 10.314 S cm^−1^ that is similar to PPy/GEL-4800s (10.389 S cm^−1^). And there is also no significant difference in the conjugate length of PPy/GEL-4800 and PPy/GEL-6000. Therefore, the longest electrochemical deposition time is 6000s.

In order to better test the conductivity of PPy/GEL and pure PPy, UV-vis absorption spectra was used in Fig. 2d. As shown in Fig. 2d, the energy band at 230 nm is mainly attributed to the π-conjugated structure of Py rings.^18^ The absorption peak in the shorter wavelength region, PPy at 337 nm, is attributed to π–π* electron transitions and represents the reduced form of the polymer backbone.^19^ In the longer wavelength region, the absorption peak at 588 nm of PPy is assigned to a charged polaron, indicating that the polymer chain is oxidized.^20^ In other words, there is a broad peak between 500 nm and 800 nm. The intensity of this peak represents the absorption of visible light, and the higher intensity of the peak represents the higher polaron content. The peak intensities of PPy/GEL-4800s and PPy/GEL-6000s are the largest and similar, indicating the best conductivity, which is consistent with the results of the previous conjugate length and four-probe resistivity tests. As showed in Fig. 2e, the PPy/GEL film is highly flexible. The black film can be cut into various letters and PPy/GEL stripe under various deformations can be used as a conductor in a circuit for lighting blue LED lamp.

**Fig. 3:**
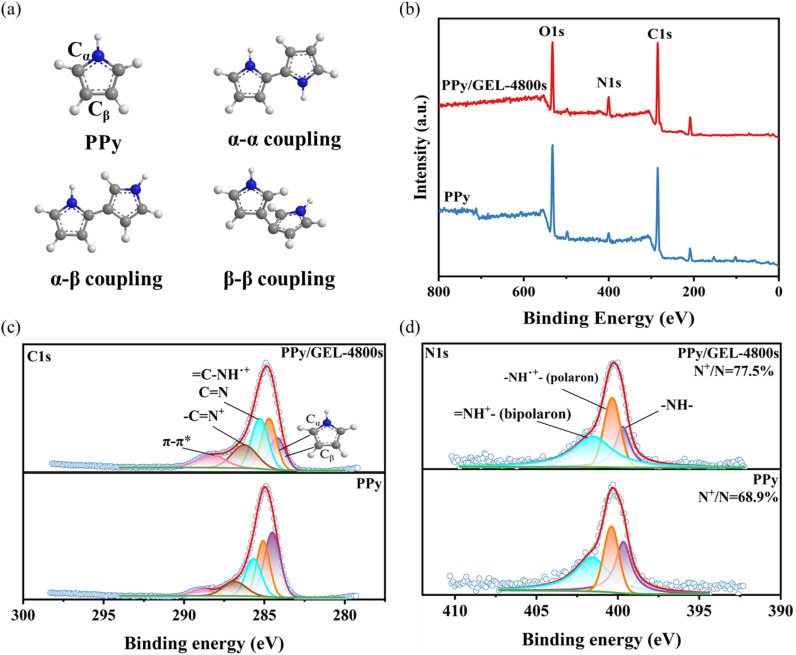
(a) Three coupling methods (α–α, α–β, β–β) of PPy. (b) XPS survey spectra of PPy/GEL and pure PPy. Fitted C 1s spectra of (c) PPy/GEL and pure PPy and (d) N 1s of PPy/GEL and pure PPy.

The XPS measurements were used to further investigate the elemental composition of pure PPy and PPy/GEL. It is well-known that there are three coupling pathways of PPy, which are α−α coupling, α−β coupling, and β−β coupling, as shown in Fig. 3a. The study verified that the α-position of the pyrrole ring is the activation site preferentially protonated and has a higher proton affinity than the N-position and the β-position.^21^ The wide region spectra of pure PPy and PPy/GEL are shown in Fig. 3b. The narrow range spectra of C1s and N1s of PPy/GEL and pure PPy are also depicted (Fig. 3c and d). Compared with the wide region spectroscopy of pure PPy, the peak of N 1s at 400 eV for PPy/GEL film spectrum has greater intensity, which means more polarons. For the electrodeposited pure PPy and PPy/GEL film, the C 1s main peak is decomposed into five lines in Fig. 3b.^22^ The lowest binding energy centered peak (main C peak) at 284.2 eV corresponds to the β-carbons of the Py ring, and the binding energy centered peak at 284.7 eV corresponds to α-carbons.^23^ Three additional peaks are identified by the deconvolution of the signal at 285.2, 286.2 and 288.3 eV, which respectively belong to the defects in doped PPy.^24^ The peak at 286.2 eV is associated to the bipolarons (–C=N^+^).^25^ The highest binding energy peak at 288.3 eV can be assigned to π–π* satellite commonly found in aromatic PPy, which is 4.1 eV higher than that of the main C peak. The content of β-carbons in PPy/GEL is less than pure PPy, so the chain structure of PPy/GEL film is more ordered. The ordered structure is beneficial to the uniform distribution of stress in the molecular chain matrix during charge–discharge cycles, thus significantly improving the cycle stability.^12^

As shown in Fig. 3d, the deconvolution of N 1s signals in the XPS spectrum gives three Gaussian components. The main N peak at 399.7 eV is attributed to the neutral N in the Py ring (–NH–).^26^ The imine-like nitrogen (=N–) is disappeared while two new high binding energy peaks appear. Two new peaks are protonation benzenoid amine (–NH˙+–), and protonation quinonoid imine (=NH + –), respectively.^27^ These two cations are usually regarded as the PPy polarons, which provide the electrochemical activity. Here, we define the polaron ratio as the ratio of the polaron signal area to the total nitrogen signal area, because the polaron pair PPy conductivity is positive.^28^ Since the polaron ratio of the PPy/GEL-4800s film is 77.5% while the pure PPy film is 68.9%, the PPy/GEL-4800s film has a higher polaron ratio than the pure PPy film, and thus the conductivity of the PPy/GEL-4800s film is better.

### 3.3 Electrochemical properties

**Fig. 4:**
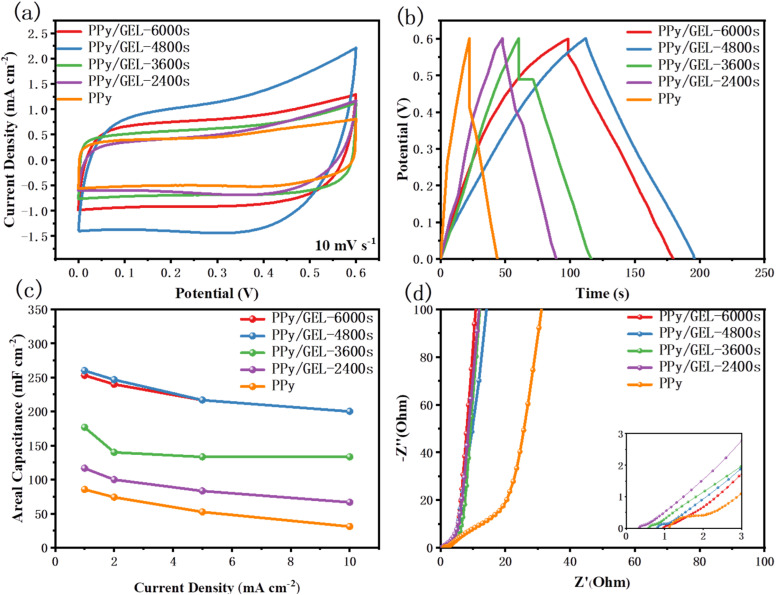
(a) CV curves of pure PPy and various PPy/GEL SCs at 10 mV s^−1^, (b) the second cycle GCD curves of pure PPy and various PPy/GEL SCs at 1 mA cm^−2^, (c) areal capacitance of pure PPy and PPy/GEL SCs at various current densities ranging from 1 to 10 mA cm^−2^ and (d) Nyquist plots.

In Fig. 4, Pure PPy and PPy/GEL electrodes were assembled into symmetrical supercapacitors (SCs) to test their electrochemical properties, respectively. By the way, wearable supercapacitors are often used in situations where accessible volume and area are limited. Therefore, delivering as much energy as possible per unit area is always a highly desirable goal for wearable supercapacitors. In the following discussion of this work, we employ areal capacitance rather than gravimetric capacitance to evaluate the performance of supercapacitors, as these parameters are important for further commercialization and practical applications.^29^

The CV results of the different samples at 10 mV s^−1^ are displayed in Fig. 4a, in which all the SCs exhibit typical quasi-rectangular profiles, suggesting their excellent capacitive behaviors.^30^ The PPy/GEL-4800s and pure PPy SCs respectively exhibited the highest and lowest CV area among the SCs, which indicate the PPy of interface deposition owning superior electrochemical performance. The GCD curves in Fig. 4b prove the same tendency that the PPy/GEL-4800s shows the longest discharging time. Therefore, 4800s is the best interface electrodeposition time. As shown in Fig. 4c, PPy/GEL SCs achieve higher rate capability and specific capacitance compared with pure PPy SC.

Besides, the rate capability and specific capacitance of PPy/GEL-4800s SC are the best. However, the rate capability and specific capacity of the pure PPy SC are the worst, with the fastest decay. This also shows that interfacial electrodeposition is superior to direct electrodeposition.

The Nyquist plots of pure PPy and PPy/GEL SCs with different deposition times from the EIS measurements are illustrated in Fig. 4d. The intercept with *x*-axis at a higher frequency presents the series resistance (*R*_s_), and the semicircle diameter indicates the charge-transfer resistance (*R*_ct_).^31^ The acquired *R*_s_ and *R*_ct_ values of the SCs are listed in Table S2. Obviously, pure PPy SCs exhibits the maximum *R*_s_ and *R*_ct_ among all the SCs, while PPy/GEL SCs own the lower *R*_s_ and *R*_ct_ value. The smaller the *R*_ct_ is, the easier the electron transfer will be. The smaller *R*_s_ indicates a faster electron transfer of the PPy/GEL electrode material at the interface between the electrode and the electrolyte. The straight line at low frequencies of PPy/GEL is closer to the *Y*-axis, indicating better diffusion of ions from the electrolyte to the electrode. All the results support that PPy/GEL has better specific capacitance performance than pure PPy electrode material. Meanwhile, with the increase of deposition time, the *R*_s_ value of PPy/GEL SCs enhanced. This may be because the longer the time, the thicker the electrode, which leads to an increase in *R*_s_.

**Fig. 5:**
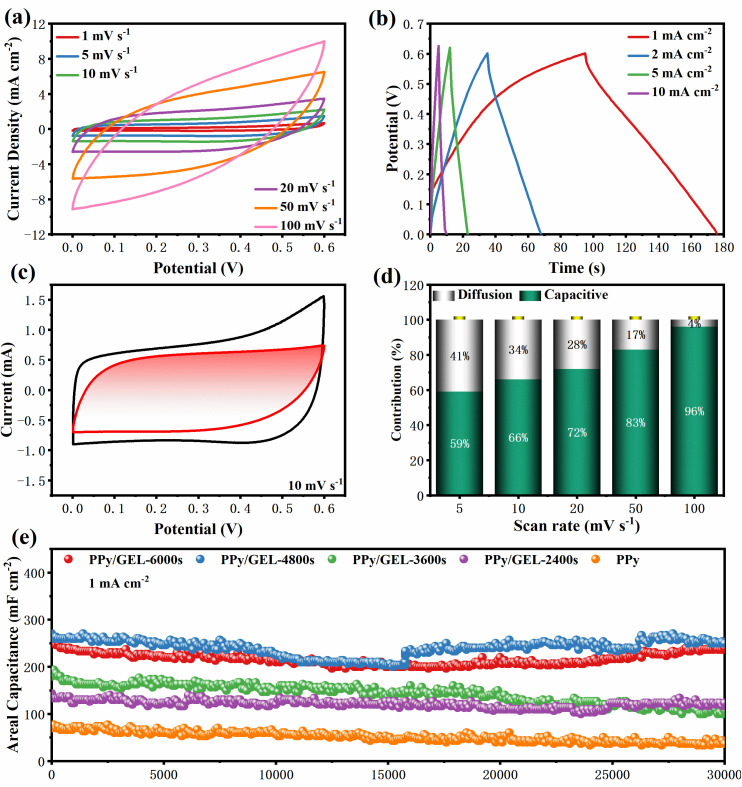
(a) CV curves and (b) GCD curves for the PPy/GEL-4800s supercapacitor. (c) CV curve of PPy/GEL-4800s at 10 mV s^−1^. (d) Capacity contribution of PPy/GEL-4800s at different scan rates. (e) Cycling performance of the PPy/GEL device at a current of 1 mA cm^−2^.

For further research electrochemical performance of PPy/GEL electrode, a symmetric supercapacitor for the two-electrode system measurements was assembled by two identical PPy/GEL-4800s electrodes. The CV curve of SC shows a spindle shape at higher scan rates, indicating that the electrode material is not the only existence of the capacitance behavior of the electric double layer^32^ (Fig. 5a). This corresponding GCD curves at different current densities show relatively good symmetry traits, indicating the reversibility of the electrode material in Fig. 5b. In order to study the enhanced electrochemical performance, the pseudocapacitance fraction was calculated (Fig. 5c and d) through the equations:^33^

a *i* = *k*_1_*v* + *k*_2_*v*_1/2_

where *k*_1_ and *k*_2_ are constants due to pseudocapacitive effects and diffusion control processes, respectively. The pseudocapacitive effect can be quantified by calculating *k*_1_*v*, and the diffusion-controlled contribution can be defined by *k*_2_*v*^1/2^. Here, when *k*_2_ is zero, we obtain the *k*_1_ value by calculating the current and scan rate ratio of the CV curve at different scan rates.

The white region occupies 34% of the CV total area, showing that the pseudocapacitance contribution is 66% at 10 mV s^−1^. The pseudocapacitance contribution increases with the increase of scan rate, and when the scan rate reachs 100 mV s^−1^, the pseudocapacitance contribution up to 96%. The charge storage process is gradually controlled by pseudocapacitance.^34^ The pseudocapacitance ratios for other sweep speeds are shown in Fig. S3.

In Fig. 5e, PPy/GEL SCs exhibit more higher areal capacitance than pure PPy SC(76 mF cm^−2^). The PPy/GEL-4800s and PPy/GEL-6000s SCs can reach a similar and high areal capacitance of 260 mF cm^−2^ and 253 mF cm^−2^, respectively. Note that the areal capacitance values are normalized by the active mass loading, which is approximately 0.5–0.7 mg cm^−2^ for PPy/GEL-4800s, facilitating direct comparison with literature. Furthermore, the capacitance of PPy/GEL SCs is only slightly reduced after 30 000 cycles of charge–discharge at 1 mA cm^−2^. The constant expansion and contraction of the polypyrrole chain results in a slight decrease of the capacitance retention during charge and discharge. Therefore, the method of interfacial electrodeposition is better than direct electrodeposition. It is probably that the existence of GelMA restricts the direction of Pyrrole monomer polymerization, which makes polypyrrole grow laterally to form a more compact film.

Beyond cycling stability, the self-discharge rate and long-term open-circuit stability (shelf life) are critical metrics for assessing the practical application of polymer-based supercapacitors. As shown in Fig. S4, the self-discharge behavior of pure PPy and PPy/GEL composite electrodes was evaluated under open-circuit conditions. Due to the faradaic pseudocapacitive mechanism, the pure PPy electrode exhibits rapid self-discharge, with the voltage decaying to approximately 30% of its initial value within 3 days. In contrast, the PPy/GEL composite electrode demonstrates significantly improved stability, maintaining about 60% of the initial voltage over the same period. This enhancement can be attributed to the confined structure provided by GelMA, which mitigates charge leakage and promotes better ion retention, thereby reducing self-discharge. These results underscore the potential of PPy/GEL electrodes for practical long-term usage in flexible electronics.

The areal capacitance of interfacial deposition is higher than that obtained for many conductive polymer-based supercapacitors, as observed in Fig. 7d, e and Table S3. The PPy/GEL-4800s SC provides an energy density of 13 µWh cm^−2^ at a power density of 600 µW cm^−2^. In addition, the energy density of up to 10 µWh cm^−2^ at a power density of 600 µW cm^−2^ in Fig. 7e highlights the advantages of these PPy/GEL SCs over many existing conductive polymer supercapacitors.

Besides, in order to further improve the area capacity of PPy/GEL-4800s SCs, we added Py monomer to GelMA, and then carried out interfacial electrodeposition.

The product is named as PPy/GEL@PPy-4800s. PPy/GEL@PPy-4800s films with different interfacial deposition times are shown in Fig. S5. As shown in Fig. S5, the formation time of PPy/GEL@PPy-4800s is shorter than that of PPy/GEL film without Py. This shows that the addition of Py monomer in GelMA is helpful to shorten the electrodeposition time.

**Fig. 6:**
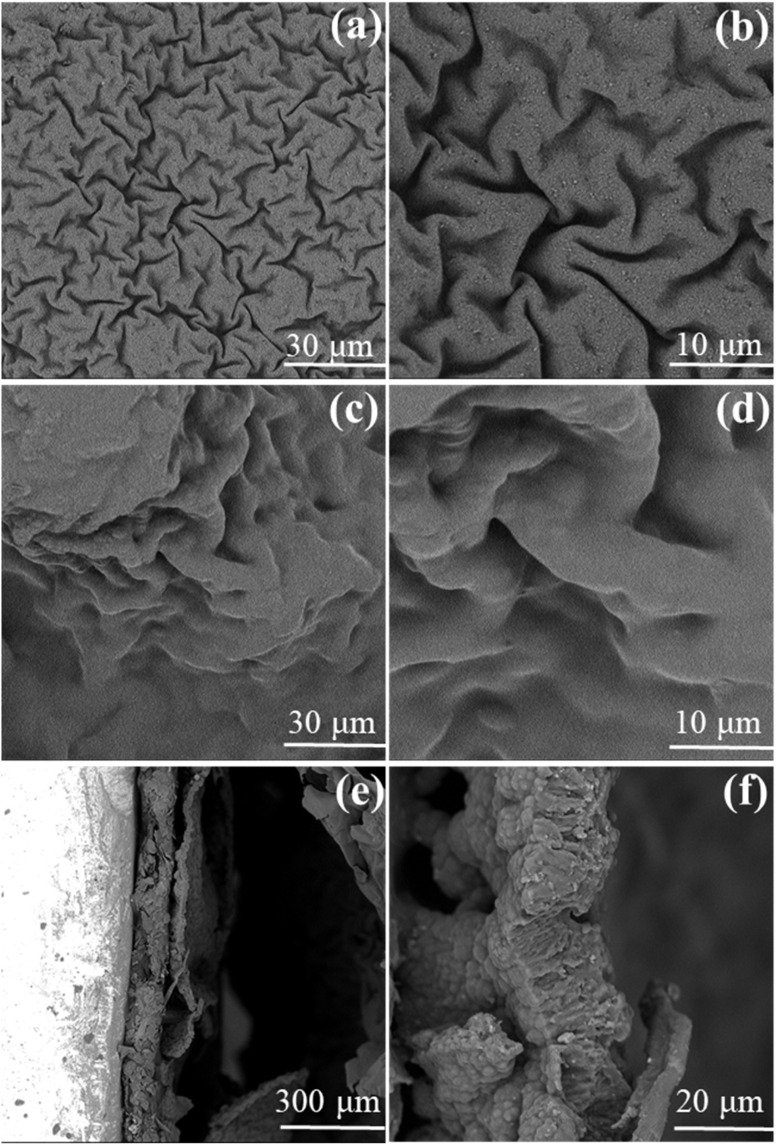
Surface SEM morphologies of PPy side of PPy/GEL@PPy-4800s at (a) low and (b) high magnifications. Surface SEM morphologies of GelMA side at (c) low and (d) high magnifications. Cross-sectional SEM images of the PPy/GEL@PPy-4800s at (e) low and (f) high magnifications.

The morphologies of PPy/GEL@PPy-4800s is shown in Fig. 6. The PPy surface remains compact and continuous (Fig. 6a and b). However, the GEL surface changed from smooth to wrinkled, which may be caused of the growth of PPy in GelMA in Fig. 6c and d. Meanwhile, the growth of PPy in GelMA is also evident from the cross-sectional view (Fig. 6e and f).

**Fig. 7:**
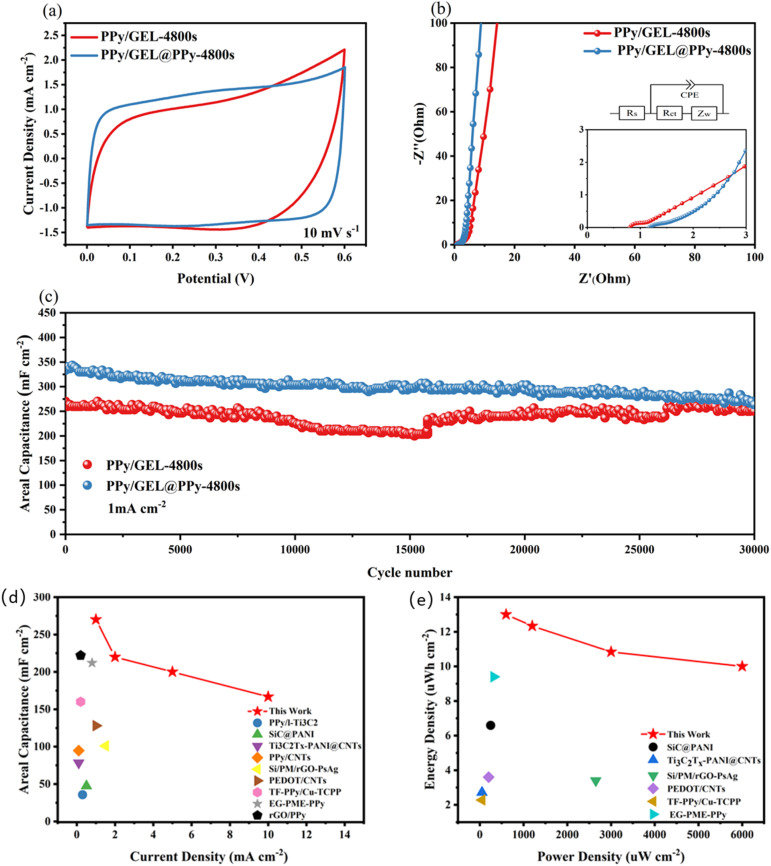
Electrochemical properties of GelMA with or without Py. (a) CV curves. (b) Nyquist plot. (c) Stability tests of SC at current density of 1 mA cm^−2^. (d) Areal capacitance in comparison with reported values. (e) Energy density in comparison with reported values.

The electrochemical performance of PPy/GEL@PPy-4800s was tested in Fig. 7. The CV curves of the two show a quasi-rectangular shape in Fig. 7a,indicating good capacitive behavior.^35^ And it is obvious that PPy/GEL@PPy-4800s exhibits larger CV curve area than PPy/GEL-4800s. This means that the addition of Py monomer to GelMA will increase the capacity of SC.

Fig. 7b is a Nyquist plot obtained from electrochemical impedance spectroscopy (EIS) detection of the PPy/GEL-4800s and PPy/GEL@PPy-4800s SCs. The vertical profile at lower frequencies reflects good capacitance due to good diffusion of ions.^36^ Therefore, the higher slope of the vertical curve of PPy/GEL@PPy-4800s SCs at lower frequencies indicates better ion diffusion. The *R*_s_ of PPy/GEL@PPy-4800s is larger than PPy/GEL-4800s SCs. It is possible that the growth of PPy in GelMA makes the film thicker and *R*_s_ increases. However, the PPy/GEL@PPy-4800s SC displays a lower *R*_ct_ value, showing the best charge transfer between the electrodes, representing its excellent capacitive properties.

Fig. 7c illustrates the stability tests of PPy/GEL@PPy-4800s and PPy/GEL-4800s SCs at current density of 1 mA cm^−2^. In Fig. 7c, the areal capacitance of PPy/GEL@PPy-4800s SC (340 mF cm^−2^) is improved significantly, comparing with PPy/GEL-4800s SC (260 mF cm^−2^). What's more, PPy/GEL@PPy-4800s SC maintains excellent charge–discharge cycling stability over 30 000 cycles. By the way, PPy/GEL-4800s SC exist an improvement in capacitance after 15 776 cycle, which may be due to the volume expansion of PPy, resulting in the electrolyte penetrating into the interior of PPy.^37^

In this study, the stable potential window of PPy/GEL SCs is 0.6 V, which was far from sufficient for the powder of daily consumer electronic products. For better application of SCs, we further integrated multiple devices in different ways (series and parallel) and characterized their corresponding electrochemical performance. Fig. S6a exhibits that when SCs are connected in parallel, the output current rises successfully. And the GCD curves of devices in parallel perform longer than the discharge time of single SC (Fig. S6b).

When SCs are connected in series, the potential window can be improved obviously in Fig. S6c. The GCD curve of the series circuit also shows a higher potential window than the single device, which is consistent with the CV curve (Fig. S6d). As show in Fig. S7, the light board can work well, which represents that our equipment has a promising business prospect. These encouraging results clearly demonstrate the potential applications of SCs.

### 3.4 Mechanical properties and swelling behavior

To substantiate the claim of improved structural integrity, swelling tests and tensile measurements were conducted on the pure PPy and PPy/GEL composite films. As demonstrated in Fig. S8(a), the PPy/GEL composite film exhibits a higher swelling ratio in the liquid electrolyte compared to the pure PPy film. This is attributed to the hydrophilic nature of the GelMA hydrogel component, which facilitates electrolyte uptake and enhances ion transport, while the confined PPy layer maintains structural coherence.

Furthermore, the tensile strength of the PPy/GEL composite film was evaluated. As shown in Fig. S8(b), the film demonstrates remarkable mechanical robustness, capable of suspending a weight of 20 kilograms without fracture. This high tensile strength ensures that the electrode can withstand repeated mechanical stress during flexible device operation, thereby guaranteeing the long-term cycling performance of the supercapacitor. The combination of controlled swelling and superior mechanical properties underscores the structural advantages of the PPy/GEL composite for durable energy storage applications.

## 4. Conclusion

In summary, we have developed a novel method for interfacial electrodeposition of polypyrrole. The GelMA coating on the FTO restricts the growth direction of PPy and makes it grow laterally into a dense film. The interfacial electrodeposited PPy/GEL film is more compact than the PPy film, and has better electrochemical properties. The PPy/GEL-4800s SC not only exhibits a high areal capacity of 260 mF cm^−2^ at 1 mA cm^−2^ but also has good rate capability, with only slight capacity fade after 30 000 cycles of charge and discharge. Furthermore, in order to further improve the electrochemical performance of PPy/GEL SC, a certain amount of Py monomer was added to gelatin for interfacial electrodeposition, and the area capacity of PPy/GEL SC obtained reached 340 mF cm^−2^ at 1 mA cm^−2^. This performance is achieved with an active mass loading of approximately 0.5–0.7 mg cm^−2^, underscoring the practicality of the electrode for flexible applications.

## Conflicts of interest

There are no conflicts to declare.

## Supplementary Material

RA-016-D5RA08151C-s001

## Data Availability

All data generated or analyzed during this study included in this manuscript and its supplementary information (SI) files are original. All data have not been used or published in any other papers. Supplementary information: the cycling performance after bending, the pseudocapacitance fraction at different scan rate, the self-discharge property, the scalability tests of supercapacitors and conductivity and charge transfer resistance of PPy/GEL based electrode. See DOI: https://doi.org/10.1039/d5ra08151c.

## References

[cit1] Zhang Y., Fu H., Xu S., Fan J. A., Hwang K.-C., Jiang J., Rogers J. A., Huang Y. (2014). A hierarchical computational model for stretchable interconnects with fractal-inspired designs. J. Mech. Phys. Solids.

[cit2] Fang Z., Peng C., Zhou Q., Liu Z. (2025). Electrocatalytic Hydrogen Peroxide Production: Advances, Challenges, and Future Perspectives. Chem. Rec..

[cit3] (a) Park S. and Jayaraman S., Smart Textiles: Wearable Electronic Systems, MRS Bull., 2003, 28(8), 585 591

[cit4] Gmucová K. (2022). Fundamental aspects of organic conductive polymers as electrodes. Curr. Opin. Electrochem..

[cit5] Liu T., Li Y. (2020). Addressing the Achilles' heel of pseudocapacitive materials: Long-term stability. InfoMat.

[cit6] Lizarraga L., María Andrade E., Victor Molina F. (2004). Swelling and volume changes of polyaniline upon redox switching. J. Electroanal. Chem..

[cit7] Ciarov J. T. (2015). Study of polypyrrole aging by XPS, FTIR and conductivity measurements. Polym. Degrad. Stab..

[cit8] Zhao Y., Zhang Z., Ren Y., Ran W., Chen X., Wu J., Gao F. (2015). Vapor deposition polymerization of aniline on 3D hierarchical porous carbon with enhanced cycling stability as supercapacitor electrode. J. Power Sources.

[cit9] Boota M., Anasori B., Voigt C., Zhao M.-Q., Barsoum M. W., Gogotsi Y. (2016). Pseudocapacitive Electrodes Produced by Oxidant-Free Polymerization of Pyrrole between the Layers of 2D Titanium Carbide (MXene). Adv. Mater..

[cit10] Lyu S., Chen Y., Han S., Guo L., Chen Z., Lu Y., Chen Y., Yang N., Wang S. (2018). Layer-by-layer assembled polyaniline/carbon nanomaterial-coated cellulosic aerogel electrodes for high-capacitance supercapacitor applications. RSC Adv..

[cit11] Khosrozadeh A., Singh G., Wang Q., Luo G., Xing M. (2018). Supercapacitor with extraordinary cycling stability and high rate from nano-architectured polyaniline/graphene on Janus nanofibrous film with shape memory. J. Mater. Chem. A.

[cit12] Huang Y., Zhu M., Pei Z., Huang Y., Geng H., Zhi C. (2016). Extremely Stable Polypyrrole Achieved *via* Molecular Ordering for Highly Flexible Supercapacitors. ACS Appl. Mater. Interfaces.

[cit13] Huang L., Yao X., Yuan L., Yao B., Gao X., Wan J., Zhou P., Xu M., Wu J., Yu H., Hu Z., Li T., Li Y., Zhou J. (2018). 4-Butylbenzenesulfonate modified polypyrrole paper for supercapacitor with exceptional cycling stability. Energy Storage Mater..

[cit14] Liang X., Zhang M., Kaiser M. R., Gao X., Konstantinov K., Tandiono R., Wang Z., Liu H.-K., Dou S.-X., Wang J. (2015). Split-half-tubular polypyrrole@sulfur@polypyrrole composite with a novel three-layer-3D structure as cathode for lithium/sulfur batteries. Nano Energy.

[cit15] Sultana I., Rahman M. M., Li S., Wang J., Wang C., Wallace G. G., Liu H.-K. (2012). Electrodeposited polypyrrole (PPy)/*para* (toluene sulfonic acid) (*p*TS) free-standing film for lithium secondary battery application. Electrochim. Acta.

[cit16] Shi X., Sun L., Li X., Wu L., Qian J., Wang J., Lin Y., Su S., Sun C., Zhang Y., Zhang Y. (2022). High-performance flexible supercapacitor enabled by Polypyrrole-coated NiCoP@CNT electrode for wearable devices. J. Colloid Interface Sci..

[cit17] Qi G., Huang L., Wang H. (2012). Highly conductive free standing polypyrrole films prepared by freezing interfacial polymerization. Chem. Commun..

[cit18] Li X.-G. (2010). Efficient and Scalable Synthesis of Pure Polypyrrole Nanoparticles Applicable for Advanced Nanocomposites and Carbon Nanoparticles. J. Phys. Chem..

[cit19] Arjomandi J., Shah A. U., Bilal S., Van Hoang H., Holze R. (2011). In situ Raman and UV-vis spectroscopic studies of polypyrrole and poly(pyrrole-2,6-dimethyl-beta-cyclodextrin). Spectrochim. Acta, Part A.

[cit20] Liu H., Xiao K., Yu M., Zhang Q., Wang D.-W. (2022). Hydrogen-bonded quasi-layered polypyrrole-tungstate complex with exceptional electrochemical capacitance over 25 000 cycles. Composites, Part B.

[cit21] Hong J., Dong Z., Chen X., Chen W., Li D., Yu F., Chen Y. (2024). α–α Coupling-Dominated PPy Film with a Well-Conjugated Structure for Superlong Cycle Life Supercapacitors. ACS Appl. Mater. Interfaces.

[cit22] Zhu M., Huang Y., Deng Q., Zhou J., Pei Z., Xue Q., Huang Y., Wang Z., Li H., Huang Q., Zhi C. (2016). Highly Flexible, Freestanding Supercapacitor Electrode with Enhanced Performance Obtained by Hybridizing Polypyrrole Chains with MXene. Adv. Energy Mater..

[cit23] Su N., Li H. B., Yuan S. J., Yi S. P., Yin E. Q. (2012). Synthesis and characterization of polypyrrole doped with anionic spherical polyelectrolyte brushes. Express Polym. Lett..

[cit24] Zhang Y., Yu Y., Xu A., Li W., Qin Y. (2022). Graphene Hydrogels Implanted onto Carbon Cloth for Polypyrrole Electrodeposition toward High-Performance Supercapacitor Electrodes. ACS Sustain. Chem. Eng..

[cit25] Song N., Gao Z., Zhang Y., Li X. (2019). B4C nanoskeleton enabled, flexible lithium-sulfur batteries. Nano Energy.

[cit26] Li X., Liu R., Xu C., Bai Y., Zhou X., Wang Y., Yuan G. (2018). High-Performance Polypyrrole/Graphene/SnCl_2_ Modified Polyester Textile Electrodes and Yarn Electrodes for Wearable Energy Storage. Adv. Funct. Mater..

[cit27] Wang J., Huang Y., Gao Y., Dai J., Sun X. (2022). The construction of carbon nanofiber composites modified by graphene/polypyrrole for flexible supercapacitors. J. Energy Storage.

[cit28] Arbizzani C., Mastragostino M., Meneghello L. (1996). Polymer-based redox supercapacitors: a comparative study. Electrochim. Acta.

[cit29] Chu X., Zhao X., Zhou Y., Wang Y., Han X., Zhou Y., Ma J., Wang Z., Huang H., Xu Z., Yan C., Zhang H., Yang W., Chen J. (2020). An ultrathin robust polymer membrane for wearable solid-state electrochemical energy storage. Nano Energy.

[cit30] Lu X., Zhang F., Dou H., Yuan C., Yang S., Hao L., Shen L., Zhang L., Zhang X. (2012). Preparation and electrochemical capacitance of hierarchical graphene/polypyrrole/carbon nanotube ternary composites. Electrochim. Acta.

[cit31] Zhou C., Zhang Y., Li Y., Liu J. (2013). Construction of high-capacitance 3D CoO@polypyrrole nanowire array electrode for aqueous asymmetric supercapacitor. Nano Lett..

[cit32] Yang S., An X., Qian X. (2021). Integrated Conductive Hybrid Electrode Materials Based on PPy@ZIF-67-Derived Oxyhydroxide@CFs Composites for Energy Storage. Polymers.

[cit33] Li L., Xu J., Shi M., He J., Jiang J., Dai K., Jiang Z., Yan C. (2022). *In situ* Raman investigation and application of MXene-stabilized polypyrrole composite for flexible aqueous batteries. Mater. Des..

[cit34] Lan L., Li Y., Zhu J., Zhang Q., Wang S., Zhang Z., Wang L., Mao J. (2023). Highly flexible polypyrrole electrode with acanthosphere-like structures for energy storage and actuator applications. Chem.–Eng. J..

[cit35] Sun J., Huang Y., Fu C., Wang Z., Huang Y., Zhu M., Zhi C., Hu H. (2016). High-performance stretchable yarn supercapacitor based on PPy@CNTs@urethane elastic fiber core spun yarn. Nano Energy.

[cit36] Chu X., Huang H., Zhang H., Zhang H., Gu B., Su H., Liu F., Han Y., Wang Z., Chen N., Yan C., Deng W., Deng W., Yang W. (2019). Electrochemically building three-dimensional supramolecular polymer hydrogel for flexible solid-state micro-supercapacitors. Electrochim. Acta.

[cit37] Sun Y., Yang Y., Fan L., Zheng W., Ye D., Xu J. (2022). Polypyrrole/SnCl_2_ modified bacterial cellulose electrodes with high areal capacitance for flexible supercapacitors. Carbohydr. Polym..

