# Supplementary material for: A flexible polypyrrole/GelMA self-supported electrode for supercapacitors by confined interfacial electrodeposition
Source: RSC Adv. 2026 Jan 19;16(5):3880–92. doi: 10.1039/d5ra08151c (PMC12814397; doi:10.1039/d5ra08151c)
Supplement: RA-016-D5RA08151C-s001 [file RA-016-D5RA08151C-s001.pdf]

Supplementary Information (SI) for RSC Advances.
This journal is © The Royal Society of Chemistry 2026

Supporting Information

A Flexible Polypyrrole/GelMA Self-supported Electrode for Supercapacitors by Confined Interfacial Electrodeposition

Gang Wang[1#], Xuezheng Chen[3#], Jiafan Guo[2], Xiaomin Su[2], Weijie Li[2], Junyi Huang[2], Wenxi Wu[2],Ting Tian[4], Feng Yu[2*]

[1]School of Materials, Guangdong Industry Polytechnic University, Guangzhou 510300, China

[2]College of Materials and Energy, Foshan University, Foshan 528225, China

[3]College of Chemical Engineering, Fuzhou University, Fuzhou 350116, China

[4]Guangdong Canosafe Pharmatech Co., Ltd., Guangzhou 510300, China

#First Author: Gang Wang and Xuezheng Chen contribute this article equally.

*Corresponding Author: Feng Yu (yuf@fosu.edu.cn)

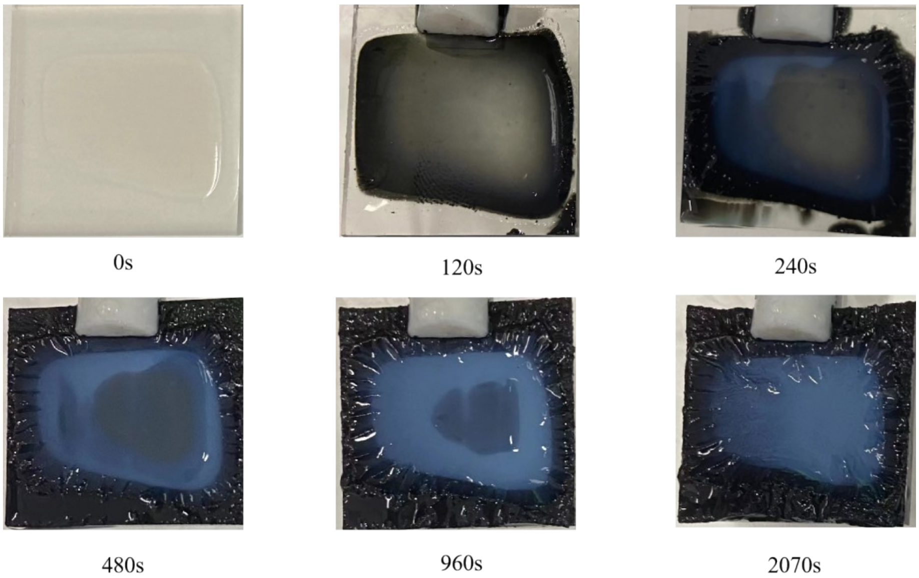

Figure S1. The photo of PPy/GEL films with different deposition time.

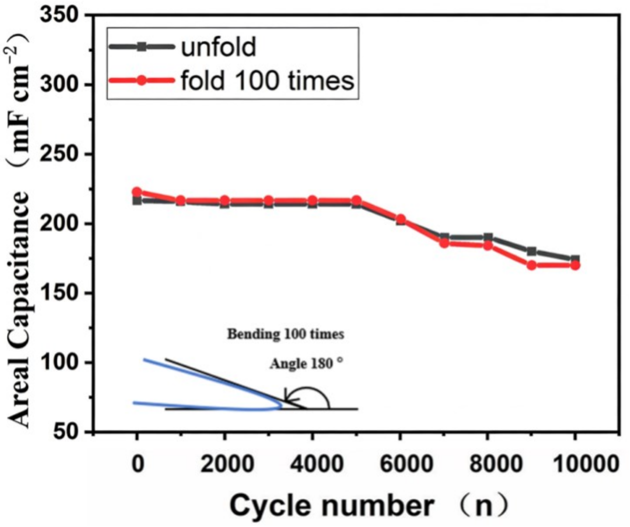

Figure S2. Long cycling performance of PPy/GEL-4800s electrode before and after 100 times 180° folding.

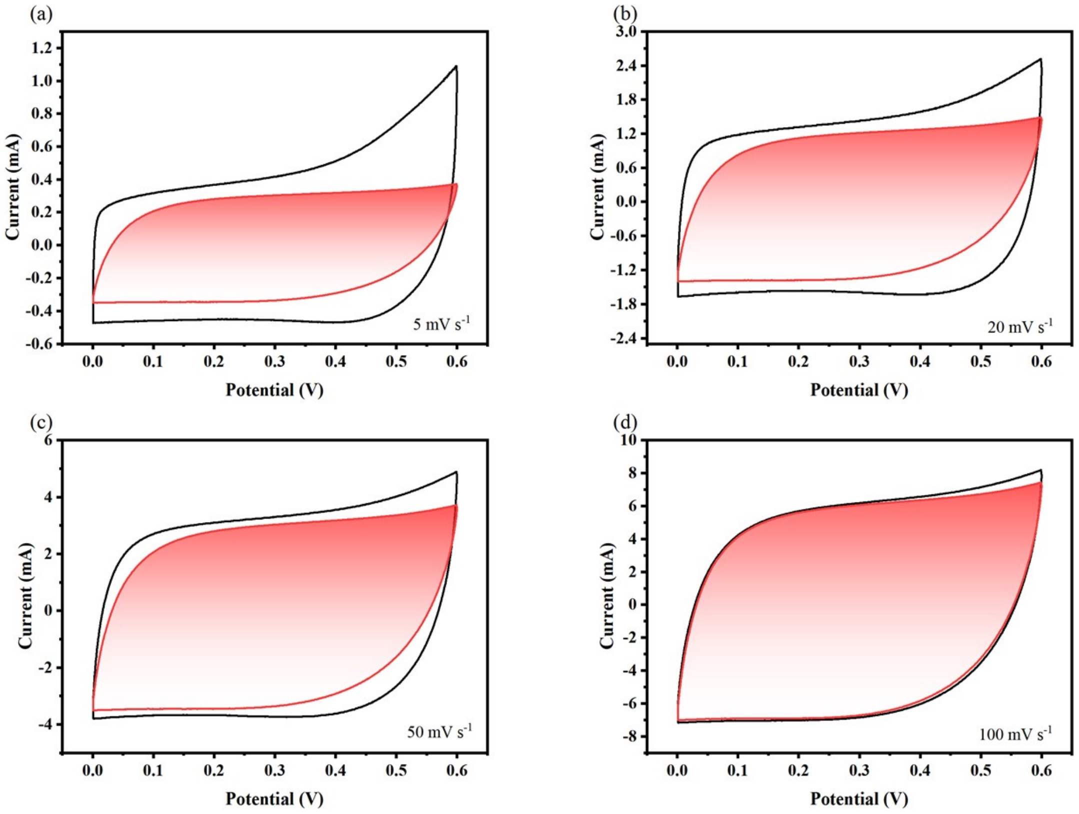

Figure S3. Pseudocapacitance fraction of (a) 5mV s^{-1},(b) 20mV s^{-1},(c) 50mV s^{-1} and (d) 100 mV s^{-1}

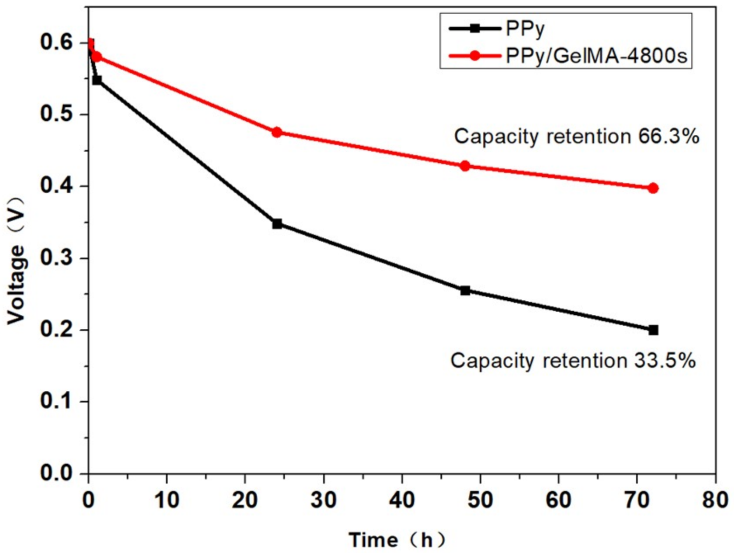

Figure S4. The self-discharge process of PPy and PPy/GelMA-4800s samples during 3 days.

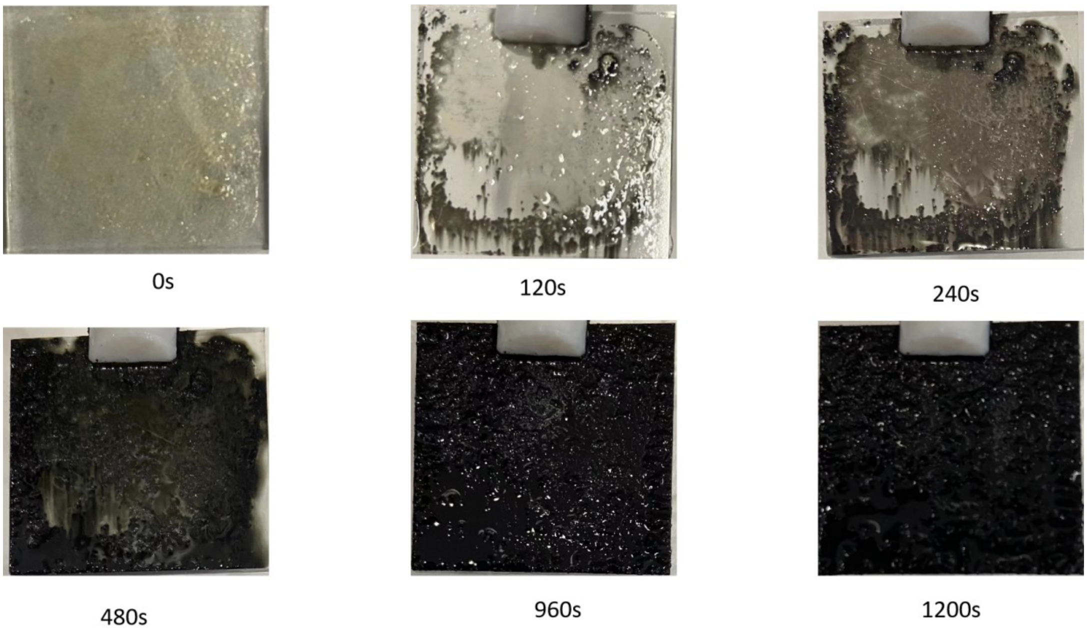

Figure S5. Pictures of PPy/GEL films containing Py in gelatin at different interfacial deposition time.

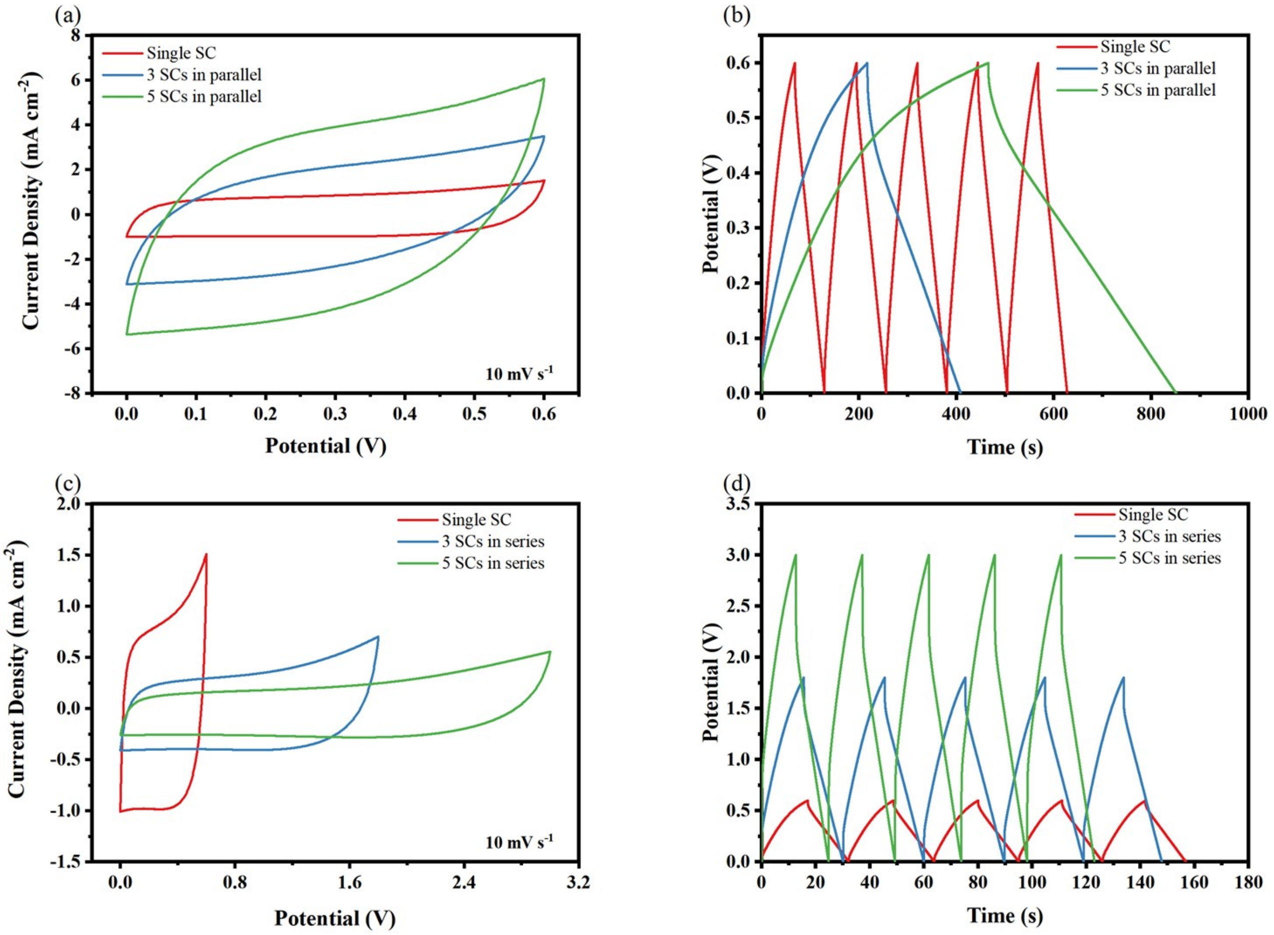

Figure S6. Scalability tests of supercapacitors made of PPy/GEL. (a) CVs of 1,3 and 5 supercapacitors in parallel. (b) GCDs of 1,3 and 5 supercapacitors in parallel. (c) CVs of 1,3 and 5 supercapacitors in series. (d) GCDs of 1,3 and 5 supercapacitors in series.

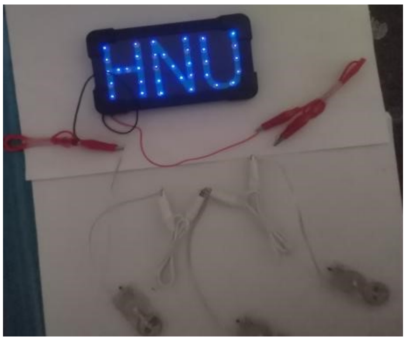

Figure S7. The 1.5V sign can be lit by connecting multiple PPy/GEL SCs in series.

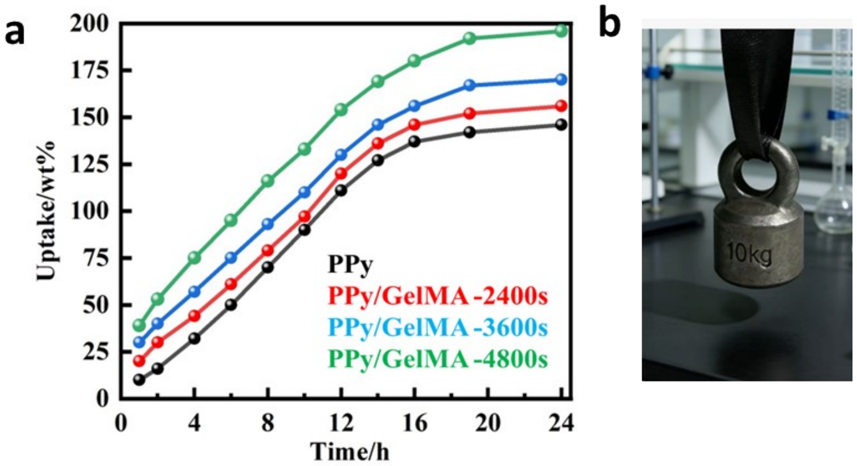

Figure S8. (a) The swelling ratio of PPy films and (b) the PPy composite film can suspend a weight of 20 kilograms.

Supporting Tables

Table S1 Conjugation Length and Conductivity of PPy/GEL and PPy

Name	I_{1580}/I_{1500}	electric conductivity
PPy/GEL-6000s	3.349	10.314
PPy/GEL-4800s	3.346	10.389
PPy/GEL-3600s	2.648	4.917
PPy/GEL-2400s	2.482	2.597
PPy	3.313	5.473

Table S2 The equivalent series resistance and charge transfer resistance of PPy/GEL SCs in different deposition time

Name	R_s	R_{ct}
PPy/GEL-6000s	0.963	21.823
PPy/GEL-4800s	0.803	7.127
PPy/GEL-3600s	0.529	7.948
PPy/GEL-2400s	0.329	11.3154
Pure PPy	1.11	120.572

Table S3 Comparison of the performance of PPy/GEL-4800s SC with the performance of other conductive polymer supercapacitors

**Supercapacitor**	**Capacitance ($mF\ cm^{-2}$)**	**Current density ($mA\ cm^{-2}$)**	**Energy density ($\mu Wh\ cm^{-2}$)**	**Power density ($\mu W\ cm^{-2}$)**	**Ref.**
**PPy/GEL-4800s SC**	**260**	**1**	**13**	**600**	**This work**
PPy/l-Ti_3C_2	35.6	0.3	NA	NA	[1]
SiC@PANI	47.48	0.5	6.59	250	[2]
$Ti_3C_2T_x$-PANI @CNTs	78.2	0.1	2.72	50	[3]
PPy/CNTs	94.7	0.1	32.8	20	[4]
Si/PM/rGO-PsAg	100.98	1.5	3.4	2652	[5]
PEDOT/CNTs	128	1	3.6	200	[6]
TF-PPy/Cu-TCPP	160	0.2	2.28	50	[7]
EG-PME-PPy	212	0.8	9.4	319.5	[8]
rGO/PPy	222	0.2	10	5000	[9]

References

[1] ZHU M, HUANG Y, DENG Q, et al. Highly Flexible, Freestanding Supercapacitor Electrode with Enhanced Performance Obtained by Hybridizing Polypyrrole Chains with MXene [J]. Advanced Energy Materials, 2016, 6(21).

[2] WANG R Y, LI W J, JIANG L, et al. Rationally designed hierarchical SiC@PANI core/shell nanowire arrays: Toward high-performance supercapacitors with high-rate performance and robust stability [J]. Electrochim Acta, 2022, 406: 139867.

[3] WANG Q Q, FANG Y S, CAO M S. Tailoring surface capacitance of Ti3C2Tx-PANI@CNTs nanoarchitecture for tunable energy storage and high-performance micro-supercapacitor [J]. Ceram Int, 2022, 48(15): 21935-44.

[4] LIU Z X, LIANG G J, ZHAN Y X, et al. A soft yet device-level dynamically super-tough supercapacitor enabled by an energy-dissipative dual-crosslinked hydrogel electrolyte [J]. Nano Energy, 2019, 58: 732-42.

[5] SHEN X J, WANG T F, WEI X Y, et al. Facile synthesis of metal oxide and conductive polymers around silicon nanowire arrays for a high-performance aqueous supercapacitor [J]. ACS Appl Energy Mater, 2022, 5(2): 2596-605.

[6] ZENG J, DONG L B, SHA W X, et al. Highly stretchable, compressible and arbitrarily deformable all-hydrogel soft supercapacitors [J]. Chem Eng J, 2020, 383: 123098.

[7] WANG W K, XU H T, ZHAO W W, et al. Porphyrin-assisted synthesis of hierarchical flower-like polypyrrole arrays based flexible electrode with high areal capacitance [J]. Chem Eng J, 2022, 428: 131089.

[8] HU Q Z, CUI S Z, SUN K J, et al. An antifreezing and thermally stable hydrogel electrolyte for high-performance all-in-one flexible supercapacitor [J]. J Energy Storage, 2022, 50: 104231.

[9] CHEN J C, WANG Y M, CAO J Y, et al. Facile Co-electrodeposition method for high-performance supercapacitor based on reduced graphene oxide/polypyrrole composite film [J]. ACS Appl Mater Inter, 2017, 9(23): 19831-42.
